# Digital Smoking Cessation With a Comprehensive Guideline-Based App—Results of a Nationwide, Multicentric, Parallel, Randomized Controlled Trial in Germany

**DOI:** 10.1093/ntr/ntae009

**Published:** 2024-01-18

**Authors:** Alexander Rupp, Stephan Rietzler, Maddalena A Di Lellis, Timo Weiland, Claudia Tschirner, Michael Kreuter

**Affiliations:** Outpatient Clinic for Pulmonary Medicine (Pneumologische Praxis im Zentrum (PiZ)), Stuttgart, Germany; Benningen, Germany; Sanero Medical GmbH, Stuttgart, Germany; novineon CRO GmbH, Tübingen, Germany; novineon CRO GmbH, Tübingen, Germany; Department of Pneumology, Mainz Centre for Pulmonary Medicine, Mainz University Medical Centre and Department of Pulmonary, Critical Care & Sleep Medicine, Marienhaus Klinikum Mainz, Mainz, Germany

## Abstract

**Background:**

Smoking tobacco implies significant health hazards. Digital cessation support can get more smokers in contact with guideline-based cessation. The objective was to test the efficacy of a guideline-based smoking cessation app (NichtraucherHelden®). The hypothesis was a significantly higher cessation rate in the intervention group.

**Methods:**

The study was a nationwide, multicentric, prospective, parallel, randomized controlled trial in Germany from November 2021 to March 2023. Recruitment took place in medical practices and by telephone via study centers. Eligible participants were adult tobacco-dependent smokers according to ICD-10 (F17.2). Randomization (1:1) was operated by a computer-generated stratified 1:1 block procedure. Intervention (IG; *n* = 336) and control group (CG; *n* = 325) were briefly advised with regard to stop smoking, IG was additionally treated with the cessation app. The primary endpoint was the self-reported 7-day-point abstinence after 6 months with an intention to treat analysis. Secondary endpoints comprised prolonged abstinence and biochemically verified abstinence. The study was registered at the German Registry of Clinical Trials (DRKS00025933, UTN U1111-1268-2181) and was approved by the competent ethics committees (leading ethic committee Berlin #Eth-52/20).

**Results:**

Three hundred thirty six participants (IG) and 325 (CG) were analyzed. Seven-day point prevalence was significantly higher in the app group (IG) (20% vs. 10%, OR 2.2 (1.4–3.4)). Additionally, the prolonged abstinence and the objective abstinence rates were significantly higher in the app group.

**Conclusions:**

The NichtraucherHelden app doubles the abstinence rate. Apps can bridge the gap between the small number of therapeutic offers and the need for modern evidence-based cessation support.

**Implications:**

The study is the first to provide evidence for the feasibility and efficacy of guideline-based digital smoking cessation provided by a smartphone app for the German statutory health insurance (SHI) system. Smoking cessation support by smartphone apps could be broadly distributed and thus bring more smokers in contact with guideline-based cessation support than to date and increase the number of successful quitters substantially.

## Introduction

### Background

As tobacco poses significant threats to the individual smoker’s health as well as to the global economy, smoking cessation and banning of tobacco products should be highly prioritized in medicine and politics. While many countries already have implemented comprehensive political measures to control tobacco consumption, Germany still is one of the poorest performers on the regularly updated tobacco control scale (TCS) in Europe,^1^ thus contributing to the high rate of currently 34.3% of adult smokers.^2^ Beside gaps in compliance with the smoke-free legislation in bars and restaurants, an incomplete ban on advertizing tobacco products, relatively low prices for tobacco products and only moderately increased tax rates over the last years, particularly limited therapeutic offers and a very restricted reimbursement policy for smoking cessation therapy seem to be accountable for the poor ranking of Germany in Europe.

Although conventional smoking cessation programs show long-term abstinence rates of 16–35%,^3^ current assumptions are that in 2021 less than 0.02% of the approximate 26 million smokers in Germany have accessed such evidence-based preventive measures supported by the statutory health insurance (SHI).^4^

Conventional smoking cessation programs include face-to-face counseling with behavioral and medication support. Digital support, however, while successfully progressing in health care in the recent past, is not yet well established in smoking cessation. In this regard, smartphone apps could be an ideal vehicle for delivering easily accessible guideline-based smoking cessation intervention. Meta-analysis has shown that digital smoking cessation therapy provided by the internet^5,6^ or by mobile phone^7^ is effective and could reach more smokers than conventional face-to-face therapy. Reported cessation rates with app-based cessation programs, mainly with automated text messaging, range from 13% to 28% with OR from about 2 to 3.^8^ The use of digital interventions for smoking cessation is therefore recommended by international and national guidelines.^9–12^ No study regarding the efficacy of smoking cessation via app exists for the German SHI.

Regarding digital support for smoking cessation, you have to consider that the quality of smoking cessation apps is very heterogenous ranging from dysfunctional apps to tracking-only apps, single-function apps, information-only apps (self-help booklets) to multifunctional apps without any information to combined apps (information and specific functions to motivate users to stop smoking) and only a minority of evidence-based apps.^13,14^ More comprehensive apps show significantly better effectiveness than apps with less content or less support.^14^

### Objective

The objective was to test the efficacy of a comprehensive guideline-based smoking cessation app (NichtraucherHelden®). Our hypothesis was that the cessation rate in the intervention group (IG) was significantly higher.

The NichtraucherHelden® app (Sanero Medical GmbH, Stuttgart, Germany) is a comprehensive, guideline-based medical app in German. It was developed on evidence-based smoking cessation therapies and current guideline recommendations by an expert panel of medical doctors. The app consists of a “core program” of nine units including the quit day and two immediate follow-up units after the quit date and an additional 76-day voluntary follow-up care period (Table S1). The app is intended to help smokers with diagnosed tobacco dependence according to the ICD-10 criteria (F17.2)^15^ aged eighteen or older.

Based on the data of a pilot study, the app was preliminarily approved by the German Federal Institute for Drugs and Medical Devices (BfArM) on July 1, 2021, to be the first digital medical app for smoking cessation. In contrast to other smoking cessation measures in Germany, the SHI fully covers the costs of the app based on new legal requirements. Expenses for supportive medication in general cannot be covered by the SHI, as current legislation in Germany is excluding the reimbursement although about 50–60% of all smokers are supposed to have a clinically relevant tobacco dependence.^16^

As no study regarding the efficacy of smoking cessation via app exists for the German SHI, we aimed to test the efficacy of the medical app. Secondary outcomes were prolonged abstinence, biochemically verified abstinence, and clinical outcome parameters (quality of life, dyspnea, and coughing). Additional explorative analysis included stratification by cessation success and by the usage of nicotine replacement therapy (NRT).

## Methods

### Trial Design

The study was a nationwide, multicentric, prospective, two-arm parallel (1:1 allocation ratio), randomized, unblinded, and controlled trial from November 2021 to March 2023. Recruitment took place in medical practices and by telephone via study centers.

### Participants

Eligible participants were adult tobacco-dependent smokers according to ICD-10 (F17.2). Participants for the study were recruited in doctor’s offices, by social media advertizing, and, because of the restrictions of the COVID-19 pandemic, by study centers via telephone and internet advertizing in Germany. Detailed inclusion and exclusion criteria are listed in Table S2. Informed consent was given by all participants before data collection. Participants received an incentive of €50 plus €20 travel expenses after finishing the study.

### Intervention

At baseline (t0), both the IG and the control group (CG) were subject to minimal intervention using semi-structured brief advice of about 3 min with regard to stopping smoking administered by a doctor. The IG then additionally received a download code for the app. No download date or start date was set. The CG was asked not to use any app or other support for the duration of the study. Follow-up was obtained 24 h (t1, only IG), 4 weeks (t2), 12 weeks (t3), and 26 weeks (t4) after the respective intervention for both groups (Figure S1). T0 and t4 were face-to-face visits in the practice or by video call, t1–t3 were online evaluations.

### Outcomes

The primary endpoint was the self-reported abstinence from tobacco products (7-day point prevalence) 6 months after the intervention that was defined according to the Society For Research on Nicotine and Tobacco (SRNT) criterion “1.b”^17^ as abstinence from all combustible and smokeless tobacco products but permitting alternative products (eg, the consumption of e-cigarettes). For this report, cessation endpoints were calculated following the Russel Standard (RS)^18^ counting all participants with missing data as smokers (intention to treat analysis, ITT).

Secondary endpoints were (SE1) prolonged self-reported abstinence (repeated 7-day point prevalence at each consecutive assessment point) after a grace period of 2 weeks, (SE2) biochemically verified abstinence (saliva cotinine after 26 weeks), (SE3) change in quality of life (QoL, measured by the physical (PCS) and the mental health component scale (MCS) of the SF12^19^), (SE4) change of the level of dyspnea (measured by the modified Medical Research Council Dyspnea Scale (mMRC)^20^) and (SE5) change of coughing (measured by modified COPD risk test (Lungenliga CH)^21^).

A cotinine test was performed only for those participants with self-reported abstinence. Saliva was collected at the doctor’s office using the Abbott Quantisal system. Alternatively, the cotinine test could be done during the video consultation under supervision and sent in. An accredited laboratory performed the saliva analysis. Participants with salivary cotinine levels < 10 ng/ml were considered non-smokers.^22^

### Sample Size

To estimate the sample size, a difference in the abstinence rate between IG and CG of 10% (18% vs. 8%) points after 6 months was assumed.^23^ To detect such a difference in abstinence rates using the exact two-sided Fisher test (*α* = 5%, allocation-ratio *N*_1_/*N*_2_ = 1) with a power of 80%, at least *n* = 384 (192 per group) complete observations are needed. The “G-Power” software^24^ was used to determine the number of cases. Studies similar to the present study have reported drop-out rates ranging from 24%^23^ to 44%.^25^ Based on these figures, a drop-out rate of 40% was assumed leading to a total of 640 participants (*n* = 320 per group).

### Randomization

Randomization (1:1) was operated using a computer-generated stratified 1:1 block procedure. Variable block lengths were used to avoid selection bias in fixed-length blocks. The randomization lists were made available to the study centers by the statistical advisor. Randomization was performed in the Electronic Data Capture (EDC) system after checking the inclusion and exclusion criteria at the respective study centers at visit t0 by local study personnel.

### Statistical Methods

The primary hypothesis of the smoking cessation rate being higher in the IG was tested with an exact Fisher test (𝛼 = 0.05). The effectiveness of the intervention regarding the secondary endpoints was tested with one-sided *z*-tests (SE1, SE2, and SE5) and one-sided *t*-tests (SE3 and SE4). The family-wise false discovery rate for these five secondary endpoints was limited to 5% applying the Holms procedure. The statistical tests were chosen by the notified body (BfArM). Complete case analyses (CCAs) for the primary endpoint and SE1 and SE2 were requested for official submission, which required over-recruiting.

Regarding the abstinence parameters, we report the results of the ITT for the primary endpoint and secondary endpoints SE1 and SE2. Missing outcome data was imputed as “non-abstinent” in line with the RS. The secondary endpoints SE3, SE4, and SE5 were analyzed with a CCA only.

All reported effect measures (risk difference (RD), odds ratio (OR), and relative risk (RR)) with corresponding 95% CI are based on univariate logistic regression models, calculated with the functions glm and risk ratio of the R-packages stats and risks, respectively. All statistical analyses were conducted in R Version 4.2.

To supplement the pre-defined tests of the primary and secondary research questions, the effect of cessation success on the health-related clinical secondary endpoints (QoL, dyspnea, and coughing) was exploratively analyzed. For this purpose, the study population was stratified into abstinent and non-abstinent participants in both groups. To assess the effect of NRT on the primary endpoint, we exploratively fitted a logistic regression model. Additionally, we compared subgroups defined by treatment group and NRT usage with respect to abstinence rates. The occurrence of withdrawal symptoms and usage of the app were analyzed descriptively.

The study was approved by the responsible ethics committees (leading ethic committee Berlin #Eth-52/20) and was registered at the German Registry of Clinical Trials (DRKS00025933, UTN U1111-1268-2181). The analysis plan was coordinated in advance with the notified body (BfArM) but not publicly registered.

## Results

**Figure 1.**
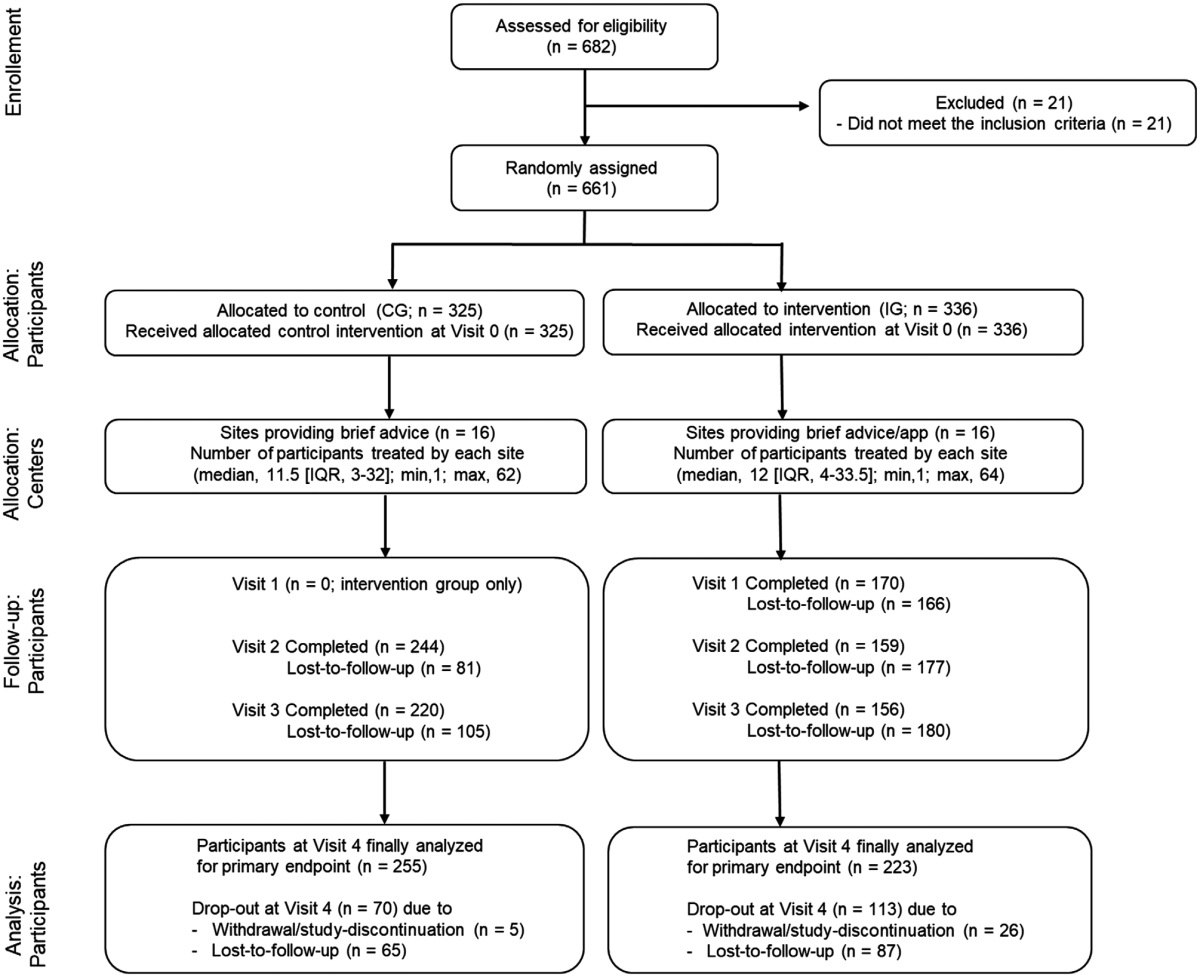
CONSORT flowchart of participants in the study. Definition “Visit 1 to 4”: All visits scheduled for data collection after the baseline/screening visit t0. Visit 1 (start of app use) is a special case, as this is only planned for participants in the intervention group. Visit 4 is decisive for primary endpoint data collection. “Visit completed”: A follow-up visit, that is, all visits except t0 (screening/baseline), is considered to have been completed, if the necessary data to determine the 7-day abstinence are available. “Lost-to-follow-up”: A participant is categorized as lost-to-follow-up at a visit if no data on 7-day abstinence is available. “Drop-out at Visit 4”: All participants for whom no data on the primary endpoint is available either because of active withdrawal or missing data on the primary endpoint at t4 because of lost-to-follow-up. “Analysis participants”: All included participants for whom data on the primary endpoint were available (7-day point prevalence after 6 months).

Between November 2021 and March 2023, 661 participants (336 IG, 325 CG) were enrolled at 17 study centers. After 6 months (t4), primary endpoint data of 478 patients (72%; IG *n* = 223 (66%) and CG *n* = 255 (78%)) were obtained. These 478 patients define the CCA population. The ITT analysis population is defined by all 661 enrolled patients, with missing data imputed as “non-abstinent.” (For details on protocol adherence and patient compliance, see the study flow chart in Figure 1.) The response rate at all visits was higher in the CG than in the app group. The trial ended regularly after reaching the planned number of participants and the last follow-up.

The main baseline data of participants are shown in Table 1 (Table S4 shows the detailed data). In brief, 61% were female and the mean age was 46 (SD 12). 21% and 19%, respectively, suffered from COPD and asthma. The average age of the participants to start smoking was 16.1 (3.2) years and the mean duration of smoking was 28 (12) years. The mean number of cigarettes smoked a day was 19 (8). 72.3% of the participants showed at least a moderate degree of tobacco addiction in the FTCD test. The mean number of quit attempts in the past was 3.6 (4.9). 87% (89% IG, 85% CG) experienced at least one withdrawal symptom of former quit attempts. Medication, mainly NRT, was used by less than one-third at former quit attempts. At baseline, 45% reported shortness of breath already when hurrying on level ground walking up a slight hill, or at minor exertion (mMRC grade ≥ 1). 57% reported coughing without having a cold at baseline and 44% had secretion when coughing. The mean PCS of SF12 at baseline was 46 (±9) points and the mean MCS was 49 (±9) points.

**Table 1.** Main Data of Participants at Baseline (t0)

	Overall	IG	CG
Participants (*n*)	661	336	325
**Sociodemographic data**			
	Mean (SD)	Mean (SD)	Mean (SD)
Age	46 (12)	46 (12)	46 (12)
Female	61.5% (402)	61.2% (205)	61.8% (197)
**Smoking-specific data**			
	Mean (SD)	Mean (SD)	Mean (SD)
Beginning of smoking (age)	16.1 (3.2)	16.2 (3.3)	16.1 (3.2)
Duration of smoking (years)	28 (12)	28 (12)	28 (12)
Nicotine dependence (FTCD)			
Very low (0–2)	9.5% (62)	9.0% (30)	10.1% (32)
Low (3–4)	18.1% (118)	17.1% (57)	19.2% (61)
Moderate (5)	14.1% (92)	14.7% (49)	13.5% (43)
Heavy (6–7)	37.0% (241)	36.6% (122)	37.4% (119)
Very heavy (8–10)	21.2% (138)	22.5% (75)	19.8% (63)
Withdrawal symptoms at previous quit attempts	87.3% (577)	89.3% (300)	85.2% (277)

### Primary Outcome

Significantly more smokers in the app group (IG) stopped smoking than in the CG: 20.2% in the IG and 10.5% in the CG reported 7-day point prevalence 26 weeks after the intervention, which results in an OR of 2.2 (1.4–3.4) for the intervention (ITT-analysis; details see Table 2). Regarding only data of participants with valid information at t4 (CCA), the cessation rate was 30% and 13%, respectively, yielding an OR of 2.9 (1.8–4.6) (Table S5).

**Table 2.** Summary of the Study Results for Self-reported 7-Day Abstinence, Prolonged Abstinence, and Objective (Biochemically Verified) Abstinence After 6 Months with Intention to Treat Analysis (ITT)

	Overall, *n* = 661	CG, *n* = 325	IG, *n* = 336	*p*-Value	RD (95% CI)	OR (95% CI)	RR (95% CI)
Abstinent (7-day)	102 (15.4%)	34 (10.5%)	68 (20.2%)	<0.001	9.8% (4.3%, 15.2%)	2.2 (1.4, 3.4)	1.9 (1.3, 2.8)
Abstinent (prolonged)	41 (6.2%)	10 (3.1%)	31 (9.2%)	<0.001	6.1% (2.5%, 9.8%)	3.2 (1.6, 7.0)	3.0 (1.5, 6.0)
Abstinent (biochem. validated)	53 (8.0%)	13 (4.0%)	40 (11.9%)	<0.001	7.9% (3.8%, 12.0%)	3.2 (1.7, 6.4)	3.0 (1.6, 5.5)

The *p*-values reported are calculated with the pre-specified tests, that is, Fishers exact test for the primary endpoint and a one-sided *z*-test for the two secondary endpoints. Additionally, the effect size measures, risk difference, odds-ratio, and risk ratio together with 95% CI are reported.

### Secondary Endpoints

The prolonged abstinence over 6 months (repeated point-prevalence) was also statistically significantly higher in the IG compared to the CG, as was the biochemically verified abstinence (Table 2). In 10% (6/59) of self-reported abstinence, the cotinine level did not confirm the abstinence, with no difference between study groups but 4 of the 6 participants with cotinine values > 10 ng/ml at t4 reported using nicotine replacement products or e-cigarettes at that point of time.

Regarding the pre-specified secondary clinical outcomes quality of life, dyspnea, and coughing only the improvement in the PCS of the SF12 was statistically significant in the IG compared to CG (Table 3).

**Table 3.** Study Results for Change in Pre-specified Secondary Clinical Endpoints from t0 to t4. All results are based on the complete case analysis (CCA). Reported *p*-values are calculated with one-sided *z*-/*t*-tests

	Overall, *n* = 478	IG, *n* = 223	CG, *n* = 255	*p*-Value
Missing	*n* = 5	*n* = 1	*n* = 4	
	Mean (SD)	Mean (SD)	Mean (SD)	
SF12 PCS	2.47 (8.03)	3.73 (7.85)	1.36 (8.05)	<0.001
SF12 MCS	–2.90 (10.38)	–2.82 (9.77)	–2.96 (10.90)	0.4
Dyspnea	–0.07 (0.67)	–0.08 (0.67)	–0.06 (0.67)	0.6
	% (*n*)	% (*n*)	% (*n*)	
Improvement in coughing	41.2% (195)	43.2% (96)	39.4% (99)	0.2

SF12: Positive values indicating improvement; dyspnea: negative values indicating improvement.

### Ancillary Exploratory Analyses

Clinical outcomes were assessed by comparing participants in both groups who achieved abstinence with non-abstinent participants in a stratification analysis (Table S6). After 6 months, both, abstinent and non-abstinent participants showed an improvement in the physical score of the SF12 (PCS). In the mental component score (MCS), non-abstinent participants even showed a deterioration while abstinent participants demonstrated moderate improvement. Abstinent participants also showed improvement on the mMRC dyspnea scale compared to non-abstinent participants and reported a much more frequent improvement in cough symptoms.

More participants of the IG reported experience of withdrawal symptoms at t2 compared to the CG (80.9% vs. 60.4%, respectively) (Figure S3). The occurrence of at least one of the given withdrawal symptoms was reported by 68.6% (269) of the participants at t2, with 35.5% (139) reported suffering from three or more of the given withdrawal symptoms (Table S7).

10.3% (68) reported utilization of cessation medication at t2, significantly more in the IG compared to the CG (14.6% vs. 5.8%, *p* < .001), mainly NRT (Table S8). Results of the logistic regression model to analyze the effect of NRT on abstinence rates are presented in Table S9. Neither the NRT main effect nor the interaction effect of NRT with treatment were significant. As the primary outcome included only tobacco products, e-cigarettes were permitted but were used by only 6.4–10.3% of participants at the different study points with no difference between the study groups, which did not interfere with the primary endpoint (Table S8).

At t1, 82% of the IG used the app at least 4 days a week (self-reported). Afterward, the frequency of app usage declined over the study period (Figure S4). Only a few participants (1–4%; t2–t4) reported the use of other support methods during the study, with no significant difference between the study groups (Table S8). After 3 months, 10% (47) reported the follow-up prescription of the app, mostly in the IG (*n* = 42 (19%) vs. *n* = 5 (2%)).

## Discussion

Smoking cessation is one of the most important therapies to reduce morbidity and mortality of smoking-related diseases and to reduce the tremendous socioeconomic costs caused by smoking every year. Because of the political framework in Germany and especially the lack of reimbursement for smoking cessation therapies until today, there are not enough therapeutic offers while the available therapies are used by only a small minority of smokers. Evidence-based digital support by an app for smokers willing to quit could bridge this gap and thus substantially increase the number of successful quitters as it would be broadly and immediately available and could deliver low-threshold access to guideline-based smoking cessation. The NichtraucherHelden medical app is the first comprehensive, guideline-based digital smoking cessation therapy in Germany. This is our report on the results of the nationwide, multicentric, prospective, randomized, and controlled trial (RCT) demonstrating significant efficacy when compared to a minimal intervention with only brief medical advice.

Our data show a significantly higher abstinence rate for the self-reported 7-day point prevalence in the app group (IG) compared to the CG (20.2% vs. 10.5%; *p* < .001) with an OR of 2.2 (1.4–3.4). A similar effect size was found regarding the secondary endpoints of prolonged and biochemically verified abstinence.

Our explanatory analysis of the effect of NRT showed a higher abstinence rate of the participants using NRT (Figure S2) (9% higher in the app group, 5% higher in the CG), although this effect was statistically not significant which was probably because of an only small number of NRT users. We nonetheless conclude that the concomitant use of NRT leads to a higher smoking cessation rate as this is also supported by meta-analyses^26^ and international guidelines.^9,10,27^ Therefore, we recommend the concomitant use of NRT while using the app.

The effect size of our study is within the range of other studies regarding digital cessation support. A preliminary version of the examined medical app already showed a significantly higher cessation rate (7-day point prevalence) of 15% after 1 year^28^ compared to cessation rates of unassisted quit attempts of 4–7%.^27^ A Cochrane review with 14 133 participants showed an RR of 1.54 (1.19–2.0) for text-based messages compared to minimal cessation support.^7^ The NICE guidelines found significant relative abstinence rates of 1.28 (1.10–1.48) to 1.38 (1.21–1.58) for digital and mobile health interventions.^11^ In a comparative study of a baseline app with a more comprehensive app, RR of 1.68 (1.25–2.28, *p* < .001; self-reported 1-month continuous abstinence) to 2.08 (1.38–3.18, *p* < .001; self-reported 3-month continuous abstinence) were found.^13^

Compared to well-known long-term cessation rates in spontaneous cessation attempts of only 4–7%,^27^ the app was able to contribute to a significant increase in the number of successful quitters. Although conventional cessation therapies in face-to-face group format show even higher cessation rates of 16–35% than cessation by app,^3^ apps have numerous advantages such as low-threshold access, the possibility of starting immediately without waiting lists, and their easy distribution to adult smokers willing to quit. Possible disadvantages compared to group therapies (eg, lower level of interaction and/or group dynamics, lower grade of individualized counseling, lower cessation rate) are outweighed by far by the disproportionately higher number of potential users.

Legal reasons requested an analysis of the clinical outcome parameters for all participants in the app group and the CG, respectively. This analysis was not able to show an effect for the app group as the study was powered to test the primary outcome and not specifically powered for the secondary outcomes. In a post hoc analysis, participants achieving abstinence showed, as expected, positive health effects, for example, improvements in the SF12 PCS, the SF12 MCS, the frequency of coughing, and the dyspnea scale. Therefore, our study was able to indicate positive health effects of abstinent participants but could not test them. Further studies should focus more on the clinical outcome parameters of abstinent participants.

The population of our study resembles the typical population of smokers in Germany according to socioeconomic and tobacco-specific data^29^ allowing the study results to be generalized for smokers in Germany. Tobacco-specific data are comparable to other smoking cessation studies applying behavioral or medical therapy.^3,26,30^ The higher rate of female participants compared to the lower smoking rate of women in the general population has already been reported by other studies on digital cessation support.^8,28,31,32^ The higher rate of COPD and asthma in the study compared to the general population^33^ is most probably caused by the number of lung practices recruited as study centers.

Six hundred sixty-one smokers participated in the study. The drop-out rate of 27% (*n* = 183) was within or rather below the expected range, even though more participants were lost to follow-up at t4 in the app group (33.6% vs. 21.5%). The reasons for the differences between the groups remain unclear as no data regarding this issue were collected in the study.

Withdrawal symptoms only occur in cessation attempts and could at the same time negatively affect the success of cessation attempts. The greater number of reported withdrawal symptoms and the higher frequency of medication use in the app group is therefore maybe because of a higher number of cessation attempts in this group but the study did not provide sufficient data to examine this.

The decrease of the app use over time was expected as the need for support diminishes in stable non-smokers and vanishes over time in participants who did not achieve abstinence.

### Strengths and Limitations of the Study

#### Strengths

The study is the first evaluation of a comprehensive guideline-based app in the context of the SHI in Germany, including 661 participants that were representative of the general population of smokers.

The results confirm the feasibility, acceptance, and efficacy of digital smoking cessation therapy by the smartphone app NichtraucherHelden®.

Therefore, the study was able to contribute to a significant improvement in medical care and therapy of dependent smokers resulting in greater abstinence rates and subsequently improvements in general health and tremendous economic savings.

#### Limitations

Although showing clearly that the examined app is a very helpful tool in smoking cessation the study was not able to show effects in the clinical outcome parameters for the whole IG as the study was not powered enough for that point and the positive health effects of successful smoking cessation could only be demonstrated in post hoc analyses.

As the study was planned under the circumstances of the COVID-19 pandemic, biochemical verification by measuring cotinine and/or the exhaled CO levels became difficult. Omitting CO measurement led to six participants at t4 showing high cotinine levels who had to be counted as (potential) smokers, although four of the six participants reported taking NRT at that point in time. This rather led to an underestimation of the evaluated efficacy of the app.

As in other studies including online visits, the rate of lost-to-follow-up was higher than in studies with face-to-face visits, which we had supposed from the very beginning and already considered in the calculation of the number of study participants. Because of missing data, there remains a little uncertainty regarding potential additional support during the study in both groups. As the intervention could not be blinded, the results may be affected by observer and information bias. Unfortunately, at the moment the app is available only in German and thus cannot be applied on an international basis.

No specific coding against any taxonomy of behavior change techniques was performed. Future studies should better focus on this topic.

In summary, the NichtraucherHelden® app is a comprehensive, guideline-based, broad and immediately available, and low-threshold digital cessation tool and showed in addition to brief advice a meaningful and significant effect on the cessation rate compared to CG participants who were given brief advice only. It could therefore help to bridge the existing gap between supply and demand for modern cessation support based on evidence and could have a sustainable effect on the abstinence rate in the general population and therefore on the future development of population-based health and socioeconomics properties. Participants achieving abstinence showed benefits regarding coughing, dyspnea, and quality of life.

## Supplementary material

Supplementary material is available at *Nicotine and Tobacco Research* online.

ntae009_suppl_Supplementary_Materials

## Data Availability

As the study data are still being evaluated by the Federal Institute for Drugs and Medical Devices, the data are not publicly available.

## Notes

German Registry of Clinical Trials (DRKS00025933, UTN U1111-1268-2181)

Leading ethic committee Berlin (#Eth-52/20).
