# Supplementary material for: Digital Smoking Cessation With a Comprehensive Guideline-Based App—Results of a Nationwide, Multicentric, Parallel, Randomized Controlled Trial in Germany
Source: Nicotine Tob Res. 2024 Jan 18;26(7):895–902. doi: 10.1093/ntr/ntae009 (PMC11190052; doi:10.1093/ntr/ntae009)
Supplement: ntae009_suppl_Supplementary_Materials [file ntae009_suppl_supplementary_materials.docx]

**Supplements**

| **Supplementary Table S1: Comprehensive content of the NichtraucherHelden® app** |
| --- |
| Preparation for the quit day (Units 1 to 8, Quit day Unit 9) |
| Coaching videos with psychoeducational content and behavioural techniques (e.g., self-monitoring, motivation to quit smoking, implementing alternative behaviour instead of smoking, rewards for not smoking, stress-reducing techniques…)  Additional information about medical support for dependent smokers  Daily tasks to be completed before the next unit is started |
| Immediately after quit day (Units 10 and 11) |
| Keeping up motivation  Coping with withdrawal symptoms and craving  Relapse prevention  Use of rewards for not smoking  Emergency unit for acute craving  Daily questions about withdrawal, difficulties, weight gain, and tailored coping strategies |
| Follow-up care (76 days) |
| Keeping up motivation  Coping with withdrawal symptoms and craving  Emergency unit for acute craving  Relapse prevention and/or therapy  Nutritional coaching (tips and recipes)  Exercise and sport coaching (fitness videos)  Daily questions about withdrawal, difficulties, weight gain, and tailored coping strategies |
| Community |
| Supportive chat with other users  Call-a-friend function |
| Cockpit function |
| Graphical tracking of smoke-free days, number of cigarettes not smoked, money saved and health benefits |

| **Supplementary Table S2: Inclusion and exclusion criteria for participation in the study** | |
| --- | --- |
| **Inclusion criteria** | **Exclusion criteria** |
| Diagnosed tobacco dependence (ICD-10: F17.2) | Concomitant dependence on alcohol, medical or illicit drugs |
| Desire to stop smoking | Acute symptomatic psychiatric state or disorder |
| Age > 18 years | Severe cognitive restrictions |
| Device available for app use | Dementia |
| Residency in Germany | Deficient language ability |
| Valid mail address | 24-h shift work |
|  | Participation in other smoking cessation measures |
|  | Use of other cessation apps |

| **Supplementary Table S3: Evaluated study items at each visit** | | | | | | |
| --- | --- | --- | --- | --- | --- | --- |
|  | t0 | t1 | t2 | t3 | t4 |  |
| App data | | | | | | |
| Frequency of use |  | IG | IG | IG | IG |  |
| Follow-up prescription |  |  |  |  | IG |  |
| Medical and sociodemographic data | | | | | | |
| Age, sex | IG/CG |  |  |  |  |  |
| Height | IG/CG |  |  |  |  |  |
| Weight | IG/CG |  |  |  | IG/CG |  |
| Marital status | IG/CG |  |  |  |  |  |
| Socioeconomic status | IG/CG |  |  |  |  |  |
| Comorbidities | IG/CG |  |  |  |  |  |
| Regular consumption of alcohol | IG/CG | IG | IG/CG | IG/CG | IG/CG |  |
| Consumption of cannabis | IG/CG | IG | IG/CG | IG/CG | IG/CG |  |
| Smoking-related data | | | | | | |
| Criteria of tobacco dependence (ICD10, F17.2) | IG/CG |  |  |  |  |  |
| Fagerström Test of Cigarette dependence (FTCD) | IG/CG |  |  |  |  |  |
| Age when started smoking | IG/CG |  |  |  |  |  |
| Mean number of smoked cigarettes | IG/CG |  |  |  |  |  |
| Number of combustible tobacco products smoked within the last 24 h, 7d, 14d* | IG/CG | IG | IG/CG  (*) | IG/CG | IG/CG |  |
| Number of e-cigarettes used within the last 24 h, 7d, 14d* | IG/CG | IG | IG/CG  (*) | IG/CG | IG/CG |  |
| Number of heat-not-burn products used within the last 24 h, 7d, 14d* | IG/CG | IG | IG/CG  (*) | IG/CG | IG/CG |  |
| Number of smokeless tobacco products consumed within the last 24 h, 7d, 14d* | IG/CG | IG | IG/CG  (*) | IG/CG | IG/CG |  |
| Number of previous quit attempts | IG/CG |  |  |  |  |  |
| Withdrawal symptoms at former quit attempts | IG/CG |  |  |  |  |  |
| Withdrawal symptoms during study |  | IG | IG/CG | IG/CG | IG/CG |  |
| Usage of other support during study |  | IG | IG/CG | IG/CG | IG/CG |  |
| Usage of cessation medication during study |  | IG | IG/CG | IG/CG | IG/CG |  |
| Willingness to quit | IG/CG | IG | IG/CG | IG/CG | IG/CG |  |
| Self-confidence regarding successful cessation | IG/CG | IG | IG/CG | IG/CG | IG/CG |  |
| Abstinence criteria | | | | | | |
| Self-report |  | IG | IG/CG | IG/CG | IG/CG |  |
| Saliva cotinine |  |  |  |  | IG/CG |  |
| Clinical outcome parameter | | | | | | |
| SF12 | IG/CG | IG | IG/CG | IG/CG | IG/CG |  |
| mMRC | IG/CG | IG | IG/CG | IG/CG | IG/CG |  |
| Coughing (modified COPD risk test) | IG/CG | IG | IG/CG | IG/CG | IG/CG |  |

| **Supplementary Table S4: Detailed data of study participants at baseline (t0)** | | | |
| --- | --- | --- | --- |
|  | Overall | IG | CG |
| Participants (n) | 661 | 336 | 325 |
| **Sociodemographic data** | | | |
|  | mean (SD) | mean (SD) | mean (SD) |
| Age | 46 (12) | 46 (12) | 46 (12) |
|  | % (n) | % (n) | % (n) |
| BMI | 26.2 (5.3) | 26.4 (5,4) | 26.1 (5.3) |
| Female | 61.5% (402) | 61.2% (205) | 61.8% (197) |
| Living with a partner | 58.9% (383) | 58.9% (196) | 59.0% (187) |
| Level of Education | | | |
| No graduation | 0.9% (6) | 0.3% (1) | 1.6% (5) |
| Grade 9 to 10 (secondary) | 51.9% (336) | 52.5% (175) | 51.1% (161) |
| Grade 11 to 13 (upper secondary) | 47.2% (306) | 47.1% (157) | 47.3% (149) |
| Monthly net income | | | |
| < 3,000 € | 70.5% (457) | 71.1% (237) | 69.8% (220) |
| 3,000 – 4,999 € | 24.0% (155) | 23.7% (79) | 24.2% (76) |
| 5,000 – 9,999 € | 5.1% (33) | 4.2% (14) | 6% (19) |
| >= 10,000 € | 0.5% (3) | 0.9% (3) | 0% (0) |
| Comorbidities |  |  |  |
| COPD | 20.9% (102) | 21.8% (55) | 19.9% (47) |
| Asthma | 18.7% (89) | 18.3% (44) | 19.2% (45) |
| Diabetes | 6.3% (26) | 5.3% (11) | 7.4% (15) |
| Hypertension | 20.2% (98) | 19.3% (47) | 21.3% (51) |
| Heart disease | 8.3% (35) | 7.5% (16) | 9.1% (19) |
| Depression | 10.9% (47) | 12.1% (27) | 9.6% (20) |
| **Health-related data** | | | |
|  | mean (SD) | mean (SD) | mean (SD) |
| SF12 (PCS) | 46 (9) | 46 (9) | 46 (10) |
| SF12 (MCS) | 49 (9) | 49 (9) | 49 (9) |
| Shortness of breath | | | |
| Only at strenuous exercise | 54.9% (357) | 56.3% (187) | 53.5% (170) |
| When hurrying or walking up a slight hill | 36.3% (236) | 35.2% (117) | 37.4% (119) |
| Lower than above exercise level | 8.7% (57) | 8.4% (28) | 9.1% (29) |
| Coughing | | | |
| Often without having a cold | 57.1% (371) | 55.7% (185) | 58.5% (186) |
| Secretion when coughing in the morning | 44.5% (289) | 44.9% (149) | 44.0% (140) |
| Coughing worsened in the last 4 weeks | 10.2% (66) | 11.2% (37) | 9.2% (29) |
| **Smoking-specific data** | | | |
|  | mean (SD) | mean (SD) | mean (SD) |
| Started to smoke (age) | 16.1 (3.2) | 16.2 (3.3) | 16.1 (3.2) |
| Duration of smoking (years) | 28 (12) | 28 (12) | 28 (12) |
| Average cigarettes per day | 19 (8) | 19 (7) | 20 (8) |
| Number of serious quit attempts | 3.6 (4.9) | 3.7 (6.0) | 3.6 (3.4) |
|  | % (n) | % (n) | % (n) |
| Medical support at previous quit attempts |  |  |  |
| Nicotine replacement therapy (NRT) | 34.3% (217) | 37.9% (122) | 30.5% (95) |
| Varenicline | 6.3% (28) | 7.4% (16) | 5.3% (12) |
| Bupropion | 3.5% (15) | 3.8% (8) | 3.1% (7) |
| Nicotine dependence (FTCD) |  |  |  |
| Very low (0 – 2) | 9.5% (62) | 9.0% (30) | 10.1% (32) |
| Low (3 – 4) | 18.1% (118) | 17.1% (57) | 19.2% (61) |
| Moderate (5) | 14.1% (92) | 14.7% (49) | 13.5% (43) |
| Heavy (6 – 7) | 37.0% (241) | 36.6% (122) | 37.4% (119) |
| Very heavy (8 – 10) | 21.2% (138) | 22.5% (75) | 19.8% (63) |
| Withdrawal symptoms at previous quit attempts | 87.3% (577) | 89.3% (300) | 85.2% (277) |
| Number of reported withdrawal symptoms at previous quit attempts |  |  |  |
| 0 | 12.9% (85) | 11.0% (37) | 14.8% (48) |
| 1 | 13.9% (92) | 13.4% (45) | 14.5% (47) |
| 2 | 26.2% (173) | 23.8% (80) | 28.6% (93) |
| 3 | 17.9% (118) | 20.5% (69) | 15.1% (49) |
| >3 | 29.2% (193) | 31.3% (105) | 27.1% (88) |
| Desire to stop smoking (VAS 1 – 10) |  | 8.6 (1.5) | 8.6 (1.6) |
| Confidence to stop successfully (VAS 1 – 10) |  | 6.5 (2.1) | 6.5 (2.1) |

| **Supplementary Table S5:** Summary of the study results for self-reported 7-day abstinence, prolonged abstinence, and objective (biochemically verified) abstinence after 6 months in the complete case analysis (CCA).  The p-values reported are calculated with the pre-specified tests. i.e., Fisher’s exact test for the primary endpoint and a one-sided z-test for the two secondary endpoints. Additionally, the effect size measures, risk difference, odds ratio, and risk ratio together with 95% CI are reported. | | | | | | | |  |
| --- | --- | --- | --- | --- | --- | --- | --- | --- |
|  | Overall, N = 478 | CG, N = 255 | IG, N = 223 | p-value | RD (95% CI) | OR (95% CI) | RR (95% CI) | |
| Abstinent (7-day) | 102 (21.3%) | 34 (13.3%) | 68 (30.5%) | < 0.001 | 17.2% (9.8%, 24.5%) | 2.9 (1.8, 4.6) | 2.3 (1.6, 3.3) | |
| Abstinent (prolonged) | 41 (8.1%) | 10 (3.5%) | 31 (13.9%) | < 0.001 | 10.4% (5.3%, 15.4%) | 4.4 (2.2, 9.7) | 3.9 (2.0, 7.9) | |
| Abstinent (biochem. validated) | 53 (12.2%) | 13 (5.5%) | 40 (20.0%) | < 0.001 | 14.5% (8.2%, 20.7%) | 4.3 (2.2, 9.7) | 3.6 (2.0, 6.6) | |

| **Supplementary Table S6: Explorative analysis of the change in health-related outcomes from t0 to t4 stratified by cessation success in a complete case analysis (CCA).** | | | |
| --- | --- | --- | --- |
|  | Overall | Abstinent | Non-abstinent |
| Participants | 478 | 102 | 376 |
|  | Mean (SD) | Mean (SD) | Mean (SD) |
| SF12 PCS | 2.5 (8.0) | 4.0 (8.0) | 2.0 (8.0) |
| SF12 MCS | -2.9 (10.4) | 1.7 (7.9) | -4.2 (10,6) |
| Dyspnea | -0,07 (0.67) | -0.28 (0.65) | -0.01 (0.67) |
|  | % (n) | % (n) | % (n) |
| Improvement in coughing | 40.8% (195) | 62.7% (64) | 34.8% (131) |
| SF12: Positive values indicating improvement; dyspnea: negative values indicating improvement | | | |

| **Supplementary Table S7: Reported withdrawal symptoms during study at T2** | | | |
| --- | --- | --- | --- |
|  | T2 | | |
|  | Overall | IG | CG |
| Reported withdrawal symptoms | 68.6% (269) | 80.9% (127) | 60.4% (142) |
| Missing data | 269 | 179 | 90 |
| Number of reported withdrawal symptoms |  |  |  |
| 0 | 31.4 (123) | 19.1 (30) | 39.6 (93) |
| 1 | 17.1 (67) | 14.0 (22) | 19.1 (45) |
| 2 | 16.1 (63) | 22.9 (36) | 11.5 (27) |
| 3 | 16.8 (66) | 21.0 (33) | 14.0 (33) |
| >3 | 18.6 (73) | 22.9 (36) | 15.7 (37) |

| **Supplementary Table S8: Medication and other support during study** | | | | | | | |
| --- | --- | --- | --- | --- | --- | --- | --- |
| Medication | | | | | | | |
|  | T2 | | T3 | | T4 | |  |
|  | IG | CG | IG | CG | IG | CG |  |
| N = | 336 | 325 | 336 | 325 | 336 | 325 |  |
| NRT | 29.9% (46) | 7.7% (18) | 23.8% (36) | 8.3% (18) | 22.2% (47) | 8.4% (21) |  |
| Missing | 182 | 91 | 185 | 107 | 121 | 76 |  |
| VAR | 0 | 0 | 0 | 0 | 0 | 0 |  |
| BUP | 2.7% (3) | 0.5% (1) | 4.2% (5) | 0.5% (1) | 4.0% (7) | 0.9% (2) |  |
| Missing | 225 | 108 | 216 | 124 | 160 | 95 |  |
| Other support | | | | | | | |
|  | IG | CG | IG | CG | IG | CG |  |
| E-cigarette | 9.4 (15) | 9.8 (24) | 6.4 (10) | 8.6 (19) | 10.3 (23) | 9.4 (24) |  |
| Missing | 177 | 81 | 180 | 104 | 113 | 70 |  |
| N = | 336 | 325 | 336 | 325 | 336 | 325 |  |
| Other support used | 2.5% (4) | 2.6% (6) | 1.3% (2) | 3.7% (8) | 3.6% (8) | 4.0% (10) |  |
| Missing | 179 | 90 | 181 | 106 | 114 | 76 |  |
|  | n = | n = | n = | n = | n = | n = |  |
| Hypnosis | 0 | 1 | 0 | 2 | 0 | 2 |  |
| Acupuncture | 0 | 0 | 0 | 0 | 0 | 0 |  |
| CBT | 1 | 0 | 0 | 1 | 0 | 0 |  |
| Another cessation app | 2 | 1 | 0 | 2 | 1 | 5 |  |
| Another programme | 1 | 4 | 2 | 5 | 3 | 2 |  |

| Supplementary Table S9: Logistic regression analysis of NRT | | | |
| --- | --- | --- | --- |
|  | OR^1^ | 95% CI^1^ | p-value |
| Treatment group |  |  |  |
| CG | -- | -- |  |
| IG | 2.04 | 1.25, 3.37 | 0.005 |
| NRT usage |  |  |  |
| NRT (no) | -- | -- |  |
| NRT (yes) | 1.56 | 0.50; 4.04 | 0.4 |
| Treatment group*NRT usage |  |  |  |
| IG*NRT (yes) | 1.06 | 0.34; 3.79 | > 0.9 |
| ^1^OR = Odds Ratio, CI = Confidence Interval | | | |

Supplementary Figure S1: Study flowchart (*IG only). Visit t1 was evaluated only for IG 24 h after finishing module 8 of the app (“Stop smoking module”). Visit t2 was obtained 4 weeks, visit t3 12 weeks and visit t4 26 weeks after the intervention respectively.

Supplementary Figure S2: Estimated proportions of responders (self-reported 7-day abstinence) by treatment-group stratified by NRT usage.

Supplementary Figure S3: Reported withdrawal symptoms at previous quit attempts (blue) and during the study (green) in % of participants.

Supplementary Figure S4: Frequency of app utilization during the study.
